# Fatal Superimposed Bacterial Sepsis in a Healthy Coronavirus (COVID-19) Patient

**DOI:** 10.7759/cureus.8350

**Published:** 2020-05-29

**Authors:** Merin Jose, Krishna Desai

**Affiliations:** 1 Internal Medicine, Saint Peter's University Hospital, New Brunswick, USA; 2 Internal Medicine, Terna Medical College, Mumbai, IND

**Keywords:** covid 19, superimposed infections

## Abstract

Coronavirus disease 2019 (COVID-19) is a highly infectious disease caused by the newly discovered coronavirus, SARS-CoV-2 (severe acute respiratory syndrome coronavirus 2). The novel coronavirus first emerged in Wuhan, China, in December 2019 and has led to a global pandemic. The virus mainly spreads through respiratory droplets from an infected person, but environmental contamination can also act as a source of infection, making social distancing an important key in containing the spread of infection. Those with underlying health conditions are more susceptible to complications such as acute respiratory distress syndrome, which can be fatal. However, healthy individuals experience a mild flu-like illness or may be asymptomatic, recuperating from the infection even without any particular intervention. We present a case of a healthy COVID positive individual, with no underlying comorbidities, who rapidly deteriorated overnight on readmission to the hospital after initial discharge and succumbed to this disease due to a superimposed bacterial infection with COVID pneumonia. This case report highlights the importance of educating COVID-19 positive patients about the precautions, as well as signs and symptoms of a superimposed bacterial infection, when their plan of care is in a home setting. It also emphasizes the potential role of checking procalcitonin levels as a part of routine laboratory investigation at initial presentation in all suspected as well as confirmed COVID-19 cases to rule out an on-going bacterial infection that can prove fatal in the course of the disease.

## Introduction

Coronaviruses can cause an array of respiratory conditions, ranging from common cold to severe acute respiratory syndrome (SARS) to the Middle East respiratory syndrome (MERS). In December 2019, a new betacoronavirus was identified, which is now known as severe acute respiratory syndrome coronavirus 2 (SARS-CoV-2) [1]. The initial clusters of pneumonia cases caused by this SARS-CoV-2 were traced in Wuhan, China [2]. In March 2020, the World Health Organization declared the outbreak to be a pandemic, with more than four million infections worldwide and still counting [3]. The spectrum of the presentation and clinical course of the infection may vary from mild to critical, with 81% of the infections being asymptomatic to mild, 14% patients developing severe disease such as dyspnea, hypoxia, or more than 50% lung involvement on imaging, and only 5% of the total infected cases progressing to a critical stage with respiratory failure and multiorgan dysfunction. Overall case fatality rate was 2.3%, with no deaths reported among non-critical cases [4]. The most common presenting symptoms are fever, dry cough (few cases with reported concurrent sputum production), dyspnea, lethargy, myalgia, and, seldom, diarrhea, hemoptysis, dysgeusia, and anosmia. The lower respiratory tract involvement can offset pneumonia, which can rapidly progress into acute respiratory distress syndrome (ARDS) and is often associated with multiorgan failure, which appears to be the most dreaded complication, as it attributes to most fatalities from the infection [1,2]. Patients with underlying comorbidities are more vulnerable to more severe complications and a poorer prognosis, as compared with healthy patients [5].

We present a case of a healthy male with no underlying comorbidities who was clinically stable on presentation and was therefore discharged for home care. Five days later, he presented again to the Emergency Department (ED) with worsening shortness of breath and diarrhea for three days and was found to have a superimposed bacterial infection. To our understanding, this caused a rapid deterioration in his clinical status and led to his early demise on the same day of his readmission. Superimposed bacterial infection has not been a frequently reported feature of this infection so far. Our emphasis from this case report is to highlight the risk of superimposed bacterial infection in COVID-19 patients. Our aim is to focus on the need to educate the patients on precautions to be taken during home care and using procalcitonin as a routine investigation for COVID-positive patients even in the absence of clinical instability [6].

## Case presentation

**Figure 1:**
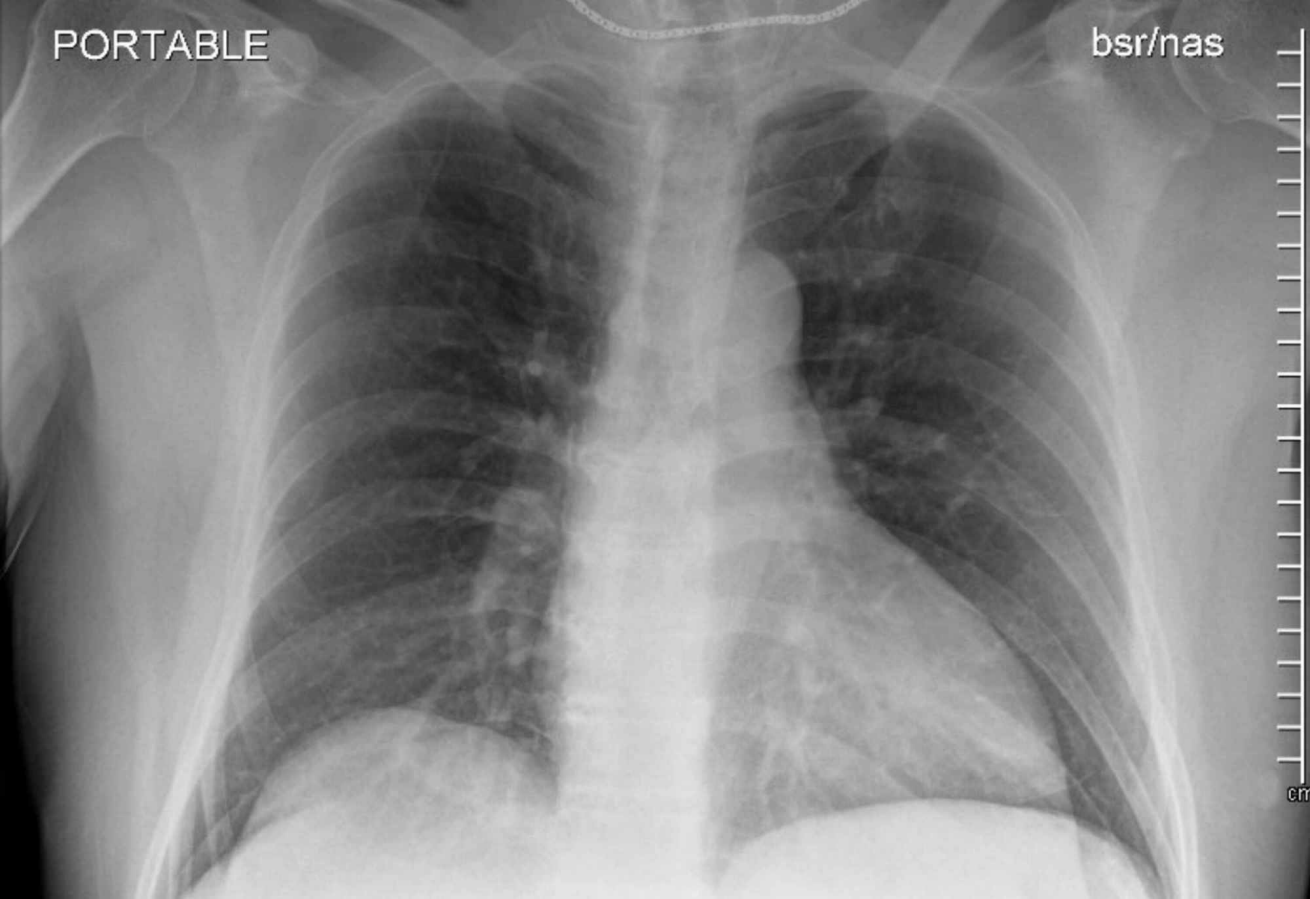
Chest X-ray with no abnormality detected on initial presentation.

A 62-year-old healthy male presented to the ED with complaints of cough, bodyache, and fever. The symptoms first appeared five days back. He had no chest pain, shortness of breath, or abdominal pain. The patient was in good health up until now, with no significant medical or surgical history. He worked as a packet distributor and was active at his baseline. He is a non-smoker and has no known addictions. He had no history of recent travel, but had a history of contact with two COVID-positive individuals (grandson and friend), and therefore COVID was suspected, for which he tested positive. On examination, he was not in distress and his vitals were stable. Chest X-ray showed clear lung and pleural spaces, a normal heart with minimally tortuous aorta, and no evidence of any active cardiopulmonary, disease, or mild hypertrophic changes in the spine, as shown in Figure 1.

Given his stable clinical condition, normal X-ray findings, and no underlying comorbidities, he was discharged home with symptomatic treatment.

Following his discharge, in the next couple of days, he developed worsening shortness of breath, spiking fever with intermittent chills, diaphoresis, and diarrhea for three days. He presented to the ED again after five days of his initial presentation for these complaints. On examination, he was hypotensive with blood pressure of 94/44 mm Hg and was hypoxic at room air saturating to 82%, which improved to 94% on 15 L of non-rebreather. He was in distress with tachypnea. He was admitted and immediately intubated, and a nasogastric tube was placed. On initial investigations, his labs were as shown in Table 1, and blood cultures were sent.

**Table 1:** Derangement in laboratory investigations on admission. WBC, white blood cell; RBC, red blood cell; BUN, blood urea nitrogen; ALP, alkaline phosphatase; AST, aspartate aminotransferase; ALT, alanine aminotransferase

	Normal reference range	Labs at admission	Notes
WBC count	4.0-11.0	19.4 cells/cumm	High with a neutrophilic predominance and low lymphocyte count
RBC count	4.40-6.20	4.94 cells/cumm	
Hemoglobin	13.0-17.0	14.7 g/dL	
Platelet count	150-400 x 10^3^/cumm	24 x 10^3^/cumm	Critically low
Serum albumin	3.2-4.6 g/dL	2.8 g/dL	Low
BUN	9-28 mg/dL	129 mg/dL	Critically high
Creatinine	0.66-1.25 mg/dL	8.30 mg/dL	High
Calcium	8.4-10.0 mg/dL	7.3 mg/dL	Low
Total bilirubin	0.1-1.2 mg/dL	8.9 mg/dL	High
ALP	56-119 U/L	223 U/L	High
AST	17-59 U/L	29	
ALT	21-72 U/L	105 U/L	High
Serum sodium	136-145 mmol/L	134 mmol/L	Low
Serum potassium	3.5-5.1 mmol/L	2.7 mmol/L	Critically low
Serum chloride	99-112 mmol/L	96 mmol/L	Low
Anion gap	<12	25	High

**Figure 2:**
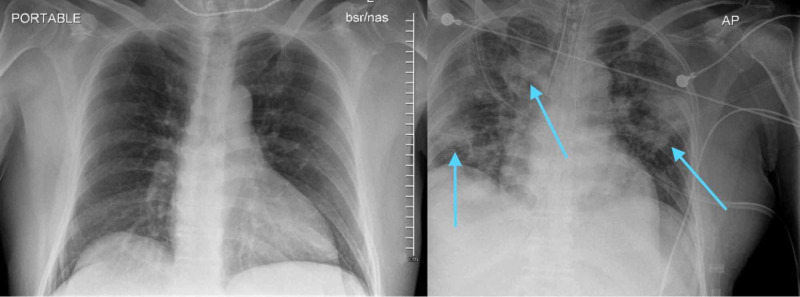
Comparison of chest X-ray changes on day 1 (left) and day 5 (right) of presentation.

Figure 2 shows chest X-rays from initial the ED visit on day 1 and subsequent admission on day 5, comparing the drastic changes in a short span of five days.

Findings of the second chest X-ray were concerning for low lung volumes and development of infectious/inflammatory process with interval development of bilateral airspace opacities, including a focal consolidation in the left upper lung. EKG showed atrial fibrillation with rapid ventricular response and non-specific T wave abnormality. Table 2 shows his arterial blood gas status at 1 hour and 9 hours after intubation.

**Table 2:** ABG at 1 hour and 9 hours post-intubation. ABG, arterial blood gas

	Normal reference range	ABG at 1 hour post-intubation	ABG at 9 hours post-intubation
pH	7.35-7.45	6.86, critically low	6.93, critically low
pCO_2_	35-48	82 mm Hg, critically high	50 mm Hg, high
pO_2_	83-108	266 mm Hg, high	133 mm Hg, high
HCO_3_-	21-28	14 mmol/L, low	10 mmol/L, low
Base excess		-22 mmol/L	-24 mmol/L
O_2_ saturation	95-99%	97%	96%

He was given 3.5 L of fluid bolus to address his hypotension. Sodium bicarbonate and norepinephrine drip was also started for pressor support. Digoxin 0.25 mg was given intravenously for atrial fibrillation and was started on amiodarone drip as options for rate control were limited, considering his hypotension. With the concern for possible superimposed bacterial infection, he was empirically started on cefepime and vancomycin intravenously (both renally dosed). He was also started on hydroxychloroquine and doxycycline. Over the next six to seven hours, he continued to worsen clinically. Blood cultures were followed up and it grew imipenem-resistant *Escherichia coli* (it was sensitive to cefazolin and piperacillin/tazobactam).

From being stable on day 1 of presentation, our patient continued to deteriorate overnight on subsequent admission. He eventually became bradycardic and was asystolic within one minute. The patient had an advance directive of "do not resuscitate" and died on the same night of admission due to septic shock with multiorgan dysfunction secondary to superimposed bacterial infection. He developed multifocal pneumonia due to COVID-19, which was possibly accelerated by superimposed *E. coli* infection or vice-versa. The possible sources of his *E. coli* bacteremia were either translocation from the bowel, given he was having diarrhea for three days, or a genitourinary source, as no symptoms pertaining to it were reported.

## Discussion

COVID-19 is a systemic disease caused by the highly pathogenic SARS-CoV-2, which led to a pandemic, globally infecting more than 4,525,497 cases and causing 307,395 deaths worldwide as of now, with the United States of America having one-third of it [3]. The incubation period for the infection is 14 days after the exposure to the virus, but most cases show symptoms by the fifth day, with an average incubation period of four days [1]. SARS-CoV-2 and previous betacoronavirus infections have overlapping clinical features.

Secondary bacterial and fungal infections are common coinfections in viral illness. Other coronaviruses outbreaks such as SARS and MERS, as well as influenza, had concurrent superimposed infections [7]. Bacterial co-infection contributed to a significant amount of mortality during previous flu pandemics in 1918 and 2009 [8]. These co-infections are associated with increased intensive care unit admissions and mortality. Superimposed bacterial infection in influenza is reported to occur in approximately 0.5% of healthy young patients and at least 2.5% of older patients. A systematic review that was performed during the 2009 pandemic reported that one out of four H1N1-infected patients had a bacterial or fungi coinfection [7]. Secondary infection was found in 50% of non-surviving COVID-19 patients [8]. Despite its commonality in prevalence and the extensive adversities it causes in SARS-CoV-2 infection, it seems to be a fairly inadequately researched topic. Furthermore, the main focus of the published papers in the literature with respect to secondary infections with other pathogens is revolving around the prevention and cross-transmission [7]. With this case report, we aim to accentuate the importance of meticulously identifying the presence of superimposed bacterial infections and attributing an adequate weightage along with other predictors for determining prognosis in a patient with SARS-CoV-2 infection.

ARDS with multiorgan failure is the most dreaded and the most common complication attributable to mortality [9]. Cardiovascular complications such as an arrhythmia, heart failure, and myocardial ischemia can also be fatal complications in patients with pneumonia [10]. Patients suffering from chronic ailments such as longstanding hypertension, diabetes, cardiovascular diseases, immunosuppressive conditions (such as malignancies, chronic lung, and kidney diseases), chronic smoking history, and advanced age comparatively had relatively poorer outcomes [10,11]. They fall under the “high-risk population” and extra caution should be maintained to keep them from acquiring the infection. According to a study, the following four predictors of higher mortality in patients with COVID-19 pneumonia were identified: (1) age ≥ 65 years, (2) preexisting concurrent cardiovascular or cerebrovascular diseases, (3) CD3+CD8+ T cells ≤ 75 cell·μL^−1^, and (4) cardiac troponin I ≥ 0.05 ng·mL^−1^ [12]. Another study suggests age, sequential organ failure assessment (SOFA) score, and D-dimer as the determinants of prognosis. The same study also highlighted that SARS-CoV-2 directly causes sepsis, even in the absence of concurrent infection with a different pathogen, but the mechanism was not well understood [10]. However, our patient with superimposed E. coli bacteremia succumbed to septic shock with multiorgan failure. He was otherwise a healthy individual in his sixties, indicating that bacterial superinfection is a risk factor even in healthy individuals without any prior hospitalization history.

A meta-analysis recommended that serial procalcitonin measurement holds an important role in predicting the development of a more critical course of the disease. Given the fact that the production of procalcitonin is ramped up in extra-thyroidal tissues in the presence of an underlying bacterial infection, its levels are also curtailed in viral infections by interferon-gamma, solidifying its correlation to complicated versus non-complicated disease processes [6].

## Conclusions

Superimposed bacterial infections are important predictors of prognosis in SARS-CoV-2 infection. It can accelerate the deterioration and can prove to be fatal despite prior optimum health of a COVID-positive patient. If the patient is clinically stable with low risk of mortality and if home care is planned, appropriate precautionary measures and warnings should be issued against bacterial and/or fungal infections and the need for immediate attention in case of any worsening of symptoms. Also, through this case, we would like to report a case of bacterial coinfection in a patient with COVID, which will help in tracking and recognizing the extent of co- or superinfection. Serial procalcitonin measurements should be routinely performed at initial presentation and thereafter to monitor and predict the prognosis of the disease.
